# Chestnut Honey Impregnated Carboxymethyl Cellulose Hydrogel for Diabetic Ulcer Healing

**DOI:** 10.3390/polym9070248

**Published:** 2017-06-27

**Authors:** Jong-Seok Park, Sung-Jun An, Sung-In Jeong, Hui-Jeong Gwon, Youn-Mook Lim, Young-Chang Nho

**Affiliations:** Advanced Radiation Technology Institute, Korea Atomic Energy Research Institute, 1266 Sinjeong-dong, Jeongeup-si, Jeollabuk-do 580-185, Korea; asj@kaeri.re.kr (S.-J.A.); sijeong@kaeri.re.kr (S.-I.J.); hjgwon@kaeri.re.kr (H.-J.G.); ymlim71@kaeri.re.kr (Y.-M.L.); jaspa@naver.com (Y.-C.N.)

**Keywords:** chestnut honey, CMC hydrogel, γ-irradiation, wound dressing

## Abstract

Honey-based wound dressings have attracted a lot of attention from modern scientists owing to their anti-inflammatory and antibacterial effects without antibiotic resistance. Such dressings also promote moist wound healing, and have been considered natural, abundant, and cheap materials for folk marketing. This study investigated the various behaviors and characteristics of chestnut honey-impregnated carboxymethyl cellulose sodium hydrogel paste (CH–CMC) as a therapeutic dressing, such as its moist retention, antibacterial activity for inhibiting the growth of *Staphylococcus aureus* and *Escherichia coli*, and the rate of wound healing in db/db mice. The results provide good evidence, suggesting that CH–CMC has potential as a competitive candidate for diabetic ulcer wound healing.

## 1. Introduction

Honey is a sweet mixture composed primarily of water, sugar (fructose and glucose, maltose), acids, proteins, minerals (mainly iron, calcium and phosphorus), and the substances and aromas of flowers, including pigments (chlorophyll derivatives), tannins, phosphate and vitamins [1,2]. It is considered a natural and effective substance for wound treatment owing to its excellent properties, such as the osmotic effect, antioxidant activity, phytochemical components, increased lymphocyte and phagocytic activity, and anti-bacterial potency [3,4]. However, honey used in any wound dressings must be carefully selected and monitored, as its quality varies based on its origin and processing [5,6,7]. In addition, antibiotic-resistant strains of bacteria should be further evaluated and determined by laboratory testing prior to clinical use [8,9,10,11].

Chestnut honey (CH) is dark colored, with a strong flavor and heavy aroma [12]. CH is one of the good candidates for wound healing. It was well known that CH has been used traditionally to treat wounds due to its high phenolic contents and flavonoids [12,13]. In addition, the tissue of chestnut plants bas been found to contain compounds such as tannins and antioxidants, which have inhibitory effects on microorganisms [14].

Hydrogel dressings have a unique property of absorption and retention of a significant amount of water, affording a moist environment for wound healing due to renewed skin and no scab formation [15]. Carboxymethyl cellulose (CMC) hydrogel has been used in drug carriers for wound care due to its ability to absorb a significant amount of water and good biocompatibility. In addition, CMC is easily crosslinked and forms hydrogel using radiation techniques, without any crosslinker or initiator [16]. In a previous study, carboxymethyl cellulose sodium (CMC) hydrogels were synthesized using a “clear” γ-irradiation technique, and these have been approved as environment friendly owing to their nontoxicity, degradability and good biological compatibility [17]. Therefore, in this work, CMC hydrogels (50 wt %) were used as a moisture and viscosity increasing agent when mixed with different concentrations of chestnut honey. We investigated the establishment of a scientific basis for ulcer wound healing application.

## 2. Experimental

### 2.1. Materials

CMC was purchased from Sigma-Aldrich Co. (St. Louis, MO, USA). Its degree of substitution was 1.2. The average molecular weight of CMC is about 2.5 × 10^5^. CH was obtained from Dongsuh Food Co., (Seoul, Korea). All aqueous solutions in this experiment were made by using deionized (DI) water, produced using a water purification system from Young Lin Instrument Co., Ltd (Anyang, Korea). All chemicals were used without any further purification.

### 2.2. Preparation of Chestnut Honey Impregnated CMC Hydrogel

To make a CH–CMC hydrogel, CH (0, 5, 10, 15, 20 wt %) and CMC powder (20 wt %) were mixed and dissolved in DI water using a planetary centrifugal mixer (Thinky Company, Tokyo, Japan) at oom temperature. The mixture was poured into a mold (40 mm × 20 mm × 2 mm) to form a dressing shape, and irradiated using gamma-rays with a total dose of 100 kGy at 10 kGy/h to produce the honey-hydrogel dressings.

### 2.3. Preparation of Chestnut Honey Impregnated CMC Hydrogel Paste (CH–CMC)

In this study, we prepared a CH–CMC hydrogel paste to verify its antibacterial activity and assess in vivo its wound healing capability. CMC powder was dissolved in DI water using a planetary centrifugal mixer (Thunky Company) at room temperature. The mixture was placed in a water bath at 75 °C for 24 h to remove air bubbles. The mixture was then poured into a square dish (125 mm × 125 mm × 20 mm), and exposed to a gamma ^60^Co source with a radiation dose (10 kGy/h) to make a crosslinking of the CMC hydrogels. Next, the CMC hydrogels were crushed into a paste state using a T25 digital ULTRA-TURRAX high-performance disperser (IKA, Staufen, Germany), Finally, CH–CMC hydrogel pastes were prepared using a CMC hydrogel (50 wt %) mixed with different concentrations of CH (5, 10, 15, 20 wt %) and DI water (45, 40, 35, 30 wt %).

### 2.4. Measurement

For measurement of the moist retention capability of the CH–CMC hydrogel, the resulting CH–CMC hydrogel (*W*_1_) was poured into a petri dish (35 × 10 mm) and dried at 37 °C until it reached a constant weight (*W*_2_). The moist retention capability of the CH–CMC hydrogel was calculated by measuring the weight loss, from the equation as follows:

Weight loss (%) = *W*_2_/*W*_1_ × 100% (1)

where *W*_1_ and *W*_2_ are the weights of the CH–CMC hydrogels in their original and dried states, respectively.

The gel content of the CH–CMC hydrogels was measured by extraction in DI water at 37 °C for 48 h and dried in an oven at 37 °C for 48 h until it reached a constant weight. The gel content (*G*_c_) was defined as

*G*_c_ (%) = (*W*_d_/*W*_i_) × 100 (2)

where *W*_d_ is the oven-dried gel weight after swelling for 48 h, and *W*_i_ is the initial weight of the dried hydrogels [18].

The degree of swelling can be described as the water absorptivity of the hydrogels. The hydrogels were immersed in distilled water for different intervals at room temperature until an equilibrium state of swelling was reached. After the excess surface fluids were removed with filter paper, the weight of the swollen gels was measured at various times. The procedure was repeated until no further weight increases were observed. The degree of swelling (*S*_w_) can be calculated as follows:

*S*_w_ (%) = [(*W*_s_ − *W*_i_)/*W*_i_] × 100 (3)

where *W*_s_ is the weight of the swollen gels at various time intervals, and *W*_i_ is the initial weight of the dried gel.

The compressive strength of the hydrogels was measured using an INSTRON 5569 (Instron Co., Norwood, MA, USA). The strength was measured using 50% compression and decompression of the hydrogels between the plates of the test machine with a crosshead speed of 10 mm/min.

The high performance liquid chromatography (HPLC) was measured using a LC 500 (GL science, Tokyo, Japan). The column was an Inertsil ODS-3 (2.1 mm × 150 mm × 3 μm). The mobile phase for HPLC consisted of water (75%) and acrylonitrile (25%). The injection volume was 5 μL and the flow rate was 1 mL/min. The wavelength for the detection was set to 425 nm. This wavelength is the first absorption band for photolysis of MGO [19].

### 2.5. Antibacterial Test

The antibacterial activity of CH–CMC paste against *Staphylococcus aureus* (*S. aureus*, ATCC 6538P) and *Escherichia coli* (*E. coli*, ATCC 25922) were studied using a petri dish (87 × 15 mm) containing 100 µL of freshly grown tryptic soy agar (TSA) seeded with 100 µL of freshly grown bacterial inoculums (10^6^ cell/mL) and pre-incubated in a 37 °C oven for 3 h. The plates were then supplemented with evenly smeared CH–CMC paste on paper (Diameter: 8 mm), and incubated at 37 °C for 24 h until a growth inhibition zone could be seen.

### 2.6. In Vivo Wound Healing Experiment

The female db/db mice (weight between 18 and 24 g, aged 5 weeks) were given an in vivo wound healing assessment. All animals were handled in strict accordance with good animal practice as defined by the standard operation procedure of the Korea Atomic Energy Research Institute (KAERI) for laboratory animal housing and husbandry. Three experimental groups, namely, those with a non-treated control wound, CMC hydrogel paste-treated wound and CH–CMC hydrogel paste-treated wound, were composed of 10 mice each. A deep round skin ulcer was inflicted on the dorsum of each subject’s body. The wound dressing was changed every 2 days, and the rate of wound contraction and microscopic observations were assessed at 0, 1, 3, 6, 9, 12, 15, and 21 days [20].

The hydrogels were evaluated for a histological analysis. Paraffin-embedded tissue blocks (6-μm-thick) were sectioned and then stained with hematoxylin and eosin (H&E). The H&E-stained sections were examined for granulation tissue formation. The thickness of the granulation tissue that formed was determined vertically at the center of each section from the surface of the granulated tissue to the margin of the dermis and the subcutis [21].

Blood samples from the mice were collected into vacutainer tubes containing ethylenediaminetetraacetic acid (EDTA) for hematology. The blood cell count was automatically calculated using a Hemavet 850 (Drew Scientific, Dallas, TX, USA).

## 3. Results and Discussion

### 3.1. Physical Properties

The moist retention capacity of the hydrogel is one of the important factors when applied to apply for wound healing [22].

**Figure 1:**
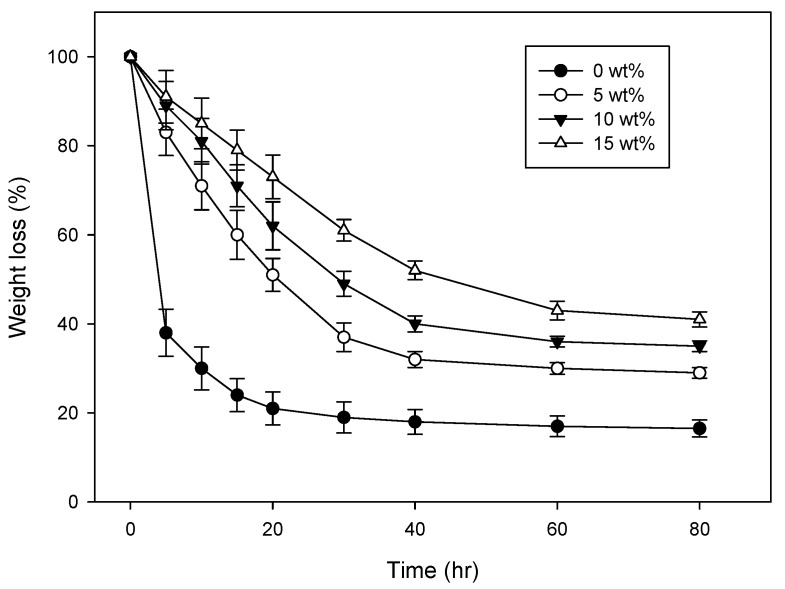
Moist retention ability of CH–CMC hydrogel with different content of honey in CH–CMC hydrogel; radiation dose is 75 kGy.

As shown in Figure 1, the moist retention capability of a CH–CMC hydrogel increases as more chestnut honey is added. The water weight decreases faster when there is a lower concentration of chestnut honey in the CH–CMC hydrogel. This result indicates that honey-based hydrogel can supply a better water environment for wound healing, which lasts for at least 2 days at body temperature. Such dressings are pappy and non-adherent, can reach a narrow deep opening, and also do not harm viable tissue or skin surrounding the wound. In addition, it can help absorb debris and excreta from inflamed wounds and can be easily cleared away.

**Figure 2:**
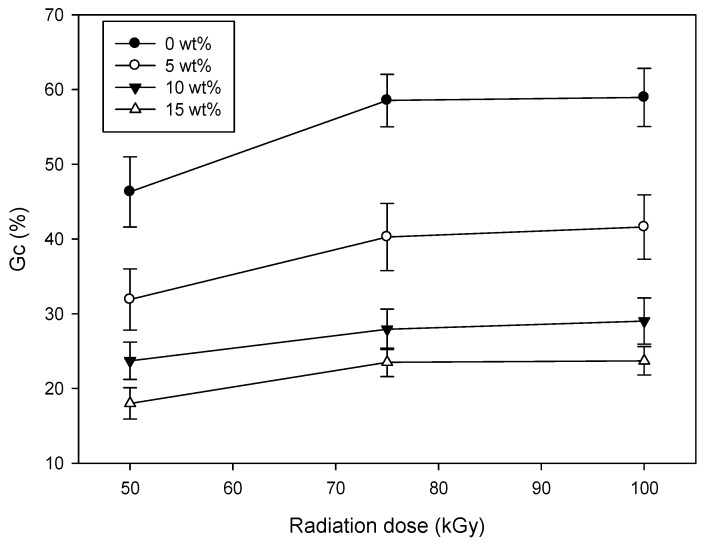
Variation of *G*_c_ with different content of honey in CH–CMC hydrogels.

Figure 2 shows the *G*_c_ as a function of CH concentration in a CH–CMC hydrogel. The *G*_c_ of the CH–CMC hydrogel increased when increasing the radiation dose. This result indicates that radiation is a useful method to make a CMC hydrogel, because gamma-ray irradiation provides energy for the formation of free radicals, accelerating the cross-linking reaction [23]. However, the *G*_c_ of the CH–CMC hydrogel decreased with an increase in the content of CH. The possible reason for this result is that the complex including sugar in CH discouraged the formation of the crosslinking structure of the CMC hydrogel [2].

**Figure 3:**
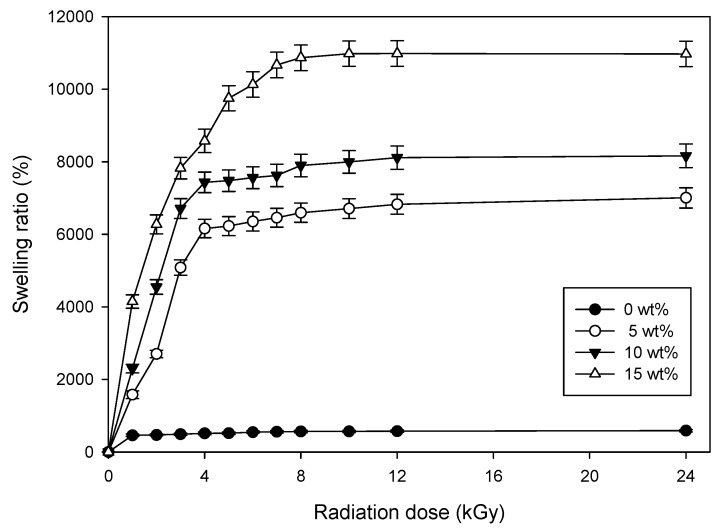
The water absorption ratio of CH–CMC hydrogels with different content of honey; radiation dose is 75 kGy.

Figure 3 shows the swelling behavior of the CH–CMC hydrogel. In this experiment, the radiation dose was 75 kGy. The swelling behavior of the CH–CMC hydrogel significantly increased with increased honey content. When comparing CH–CMC hydrogel (15 wt %) to neat CMC hydrogel (0 wt %), the difference in swelling ratio was indicated to be multiplied more than tenfold. These results indicate that CH–CMC hydrogel can provide a better moisture environment, and absorb wound exudates for wound healing. 

**Figure 4:**
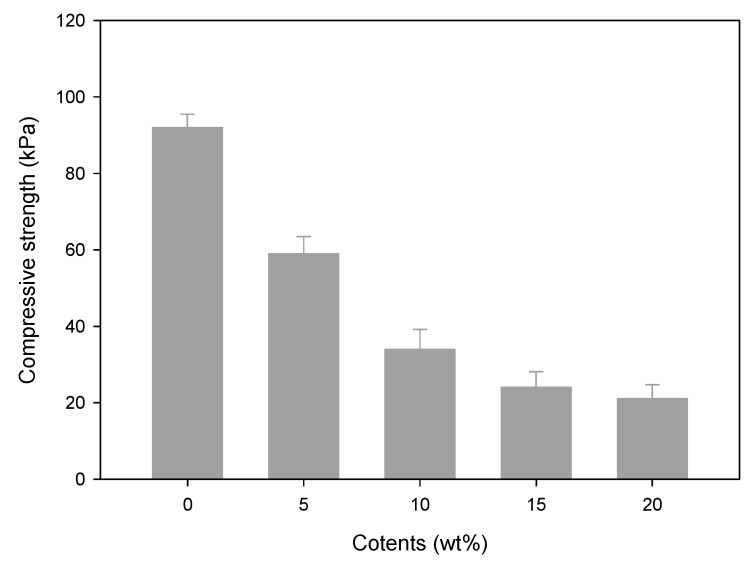
Compressive strength of CH–CMC hydrogels with different content of honey; radiation dose is 75 kGy.

Figure 4 shows the compressive strength for the CH–CMC hydrogel with the contents of honey. The compressive strength decreased with an increase in honey content. The compressive strength of pristine CMC hydrogel (0 wt %) was measured at 91 kPa, whereas that of CH–CMC hydrogel (20 wt %) was measured at 22 kPa. In general, the compressive strength of the hydrogel was proportional to the *G*_c_. In fact, the compressive strength of the CMC hydrogel substantially decreased owing to the presence of CH as compared with a pristine CMC hydrogel.

### 3.2. Antibacterial Activity

In this study, we prepared a CH–CMC hydrogel paste in order to verify its antibacterial activity and in vivo wound healing assessment. CMC hydrogels were prepared using a gamma-ray. Next, the CMC hydrogels were crushed into a paste. Finally, CH–CMC hydrogel pastes were prepared using CMC hydrogel (50 wt %) mixed with different concentrations of CH (5, 10, 15, 20 wt %) and DI water (45, 40, 35, 30 wt %).

**Figure 5:**
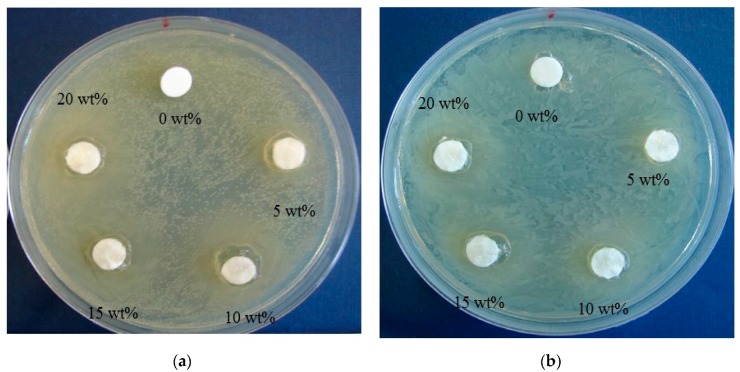
Antibacterial image of CH–CMC hydrogels with different content of honey against *S. aureus* (**a**) and *E. coli* (**b**).

In this work, the antibacterial effect of the CH–CMC hydrogel pastes against *S. aureus* and *E. coli* strains was investigated. The growth inhibition was determined using paper disk diffusion assays. Figure 5 and Table 1 show the growth inhibition zones of different CH–CMC samples on Tryptic Soy agar (TSA) plates. The results show that the CH–CMC hydrogels have an effect on inhibiting and killing these two types of bacteria. In particular, obvious inhibition zones appear against *S. aureus*. Some researchers have reported that various parameters of honey, such as pH, H_2_O_2_, and osmotic pressure, may aid in its antibacterial actions, but have a stinging effect during the therapeutic process [24]. In addition, it has been documented that the pronounced antibacterial activity of honey originated from the methyglyoxal (MGO) [24,25].

**Figure 6:**
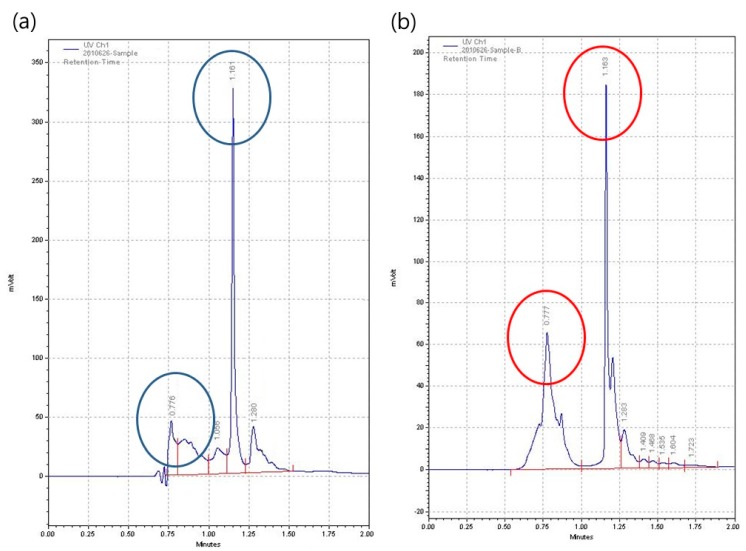
HPLC peaks of (**a**) methylglyoxal (MGO) and (**b**) chestnut honey (CH).

Figure 6 shows the HPLC profiles of MGO and CH. The spectra of MGO correlation with the antibacterial activity are characterized by a main peak at 1.161 min and a second peak at 0.776 min. When applied to CH, a similar result was obtained. The main peak of CH was centered at 1.163 min, and the peak intensity was about 180 mV. In addition, the second peak was observed at 0.777 min. It was demonstrated that the high MGO levels are correlated with antibacterial activity against a range of bacterial species [26,27,28,29,30]. In addition, the correlation between the MGO levels and the antibacterial activity, it has been demonstrated that MGO is responsible for the nonperoxide antibacterial activity of honey, and that low MGO levels don’t affect the activity against bacteria [31]. Hence, the CH–CMC hydrogel having an abundant MGO ingredient inhibited and killed *S. aureus* bacteria and Gram-negative *E. coli* bacteria. 

### 3.3. In Vivo Wound Healing Assessment

The female db/db mice (weight between 18 and 24 g, aged 5 weeks) were given an in vivo wound healing assessment. Three experimental groups, namely, those with a non-treated control wound, CMC hydrogel paste-treated wound, and CH–CMC hydrogel paste-treated wound, were composed of 10 mice each. A deep round skin ulcer was inflicted on the dorsum of each subject’s body.

**Figure 7:**
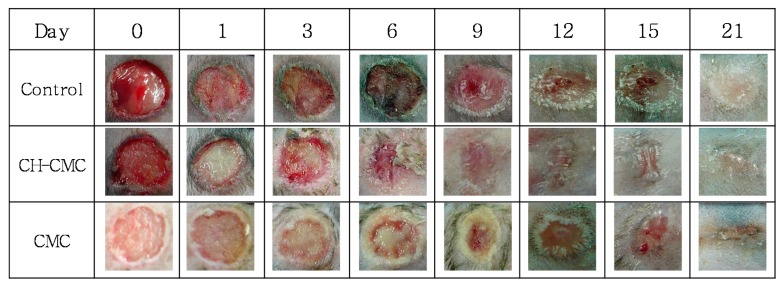
Photographs of wound healing observation by dates; control (non-treated wound), CH–CM (CH–CMC hydrogel-treated wound), CMC (CMC hydrogel-treated wound).

Figure 7 shows the photographs of wound recovery during the evaluation period. Compared with the control (non-treated wounds), CMC (20 wt %) hydrogel paste-treated wounds and CH–CMC (20 wt %) hydrogel paste-treated wounds were contracted at the highest rate. In particular, the CH–CMC dressings significantly stimulated the rate of healing for a surgical wound. On the contrary, the untreated control wounds became infected and inflamed at day 6, accompanied with excreta and bad odor. The CH–CMC dressing hastened the healing rate by accelerating the wound contraction compared with wounds treated only with CMC dressings. They were both cured and debrided in moist environments with less scab formation. In particular, the CH–CMC dressings showed a more significant acceleration of dermal repair than other trials. On day 21, wounds treated with CH–CMC dressings were almost healed.

**Figure 8:**
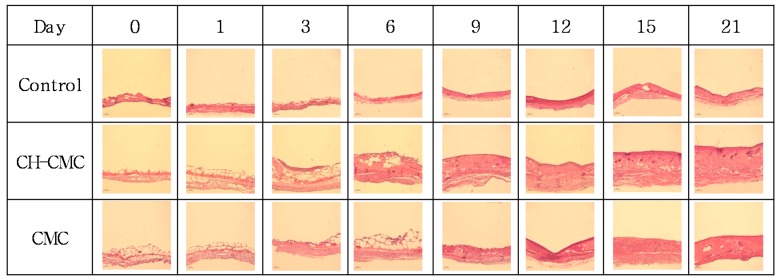
Histology image of granulation tissue in the wound by dates on consecutive days; control (non-treated wound), CH–CM (CH–CMC hydrogels treated wound), CMC (CMC hydrogels treated wound).

The treatment tendency of ulcers was studied by examining the histology. Figure 8 shows the change in granulation tissue formation. The histology evaluation demonstrated that there was a significant acceleration of dermal repair in wounds treated with CH–CMC dressings. There were no significant differences in the granulation tissue formation among the three groups on the first day. However, compared with the control (non-treated wounds) and CMC (CMC dressing-treated wounds), the thickness of granulation tissue in wounds treated with the CH–CMC dressing began to be significantly enhanced on the third day. The granulation tissue in wounds treated with CMC dressings grew slowly until day 9, and a rapid growth began from day 15. On the other hand, there was not much difference from the thickness of the granulation tissue of the control between day 0 and day 21. This result indicates that the CMC hydrogel supplied a greater water environment for wound healing, and that the CH in CMC hydrogel further accelerated the granulation tissue formation.

**Figure 9:**
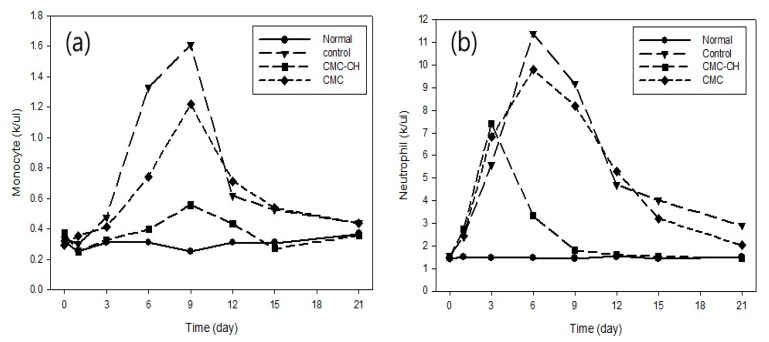
(**a**) Monocyte and (**b**) neutrophil in wound healing; Normal (harmless mice) control (non-treated wound), CH–CM (CH–CMC hydrogels treated wound), CMC (CMC hydrogels treated wound).

Figure 9 shows the volume of monocyte and neutrophil in wound healing. Because the amount of neutrophil in mice decreased at this point, the amount of monocyte began to increase. Generally, neutrophil, which is responsible for preventing the invasion of bacteria and eliminating necrotic tissue, dwindled to nothing due to the appearance of the monocyte. The amount of neutrophil in wounds treated with CH–CMC dressing increased significantly on the third day, and then began to decrease. However, the amount of neutrophil with the control (non-treated wounds) and CMC (CMC dressings treated wounds) increased on the sixth day, and then began to decrease. In addition, the difference in the amount of monocyte in normal mice and those with CH–CMC dressing-treated wounds was much less significant than the control and CMC. It should be noted that CH has a good capability to prevent the invasion of bacteria and treat wounds.

## 4. Conclusions

In this study, we adequately utilized and combined the advantages of CH and CMC hydrogels using a simple and “green” method. CH–CMC hydrogels have an effect on inhibiting and killing bacteria. In addition, the resulting effect of the sample on wound healing by an in vivo experimental assessment was focused on, and showed good evidence for application.

## Figures and Tables

**Table 1:** Antibacterial activity CH–CMC hydrogels; (-) without inhibition, (+) *d*_r_ = 1.0 mm, (++) *d*_r_ = 2.0 mm, (+++) *d*_r_ = 3.0 mm, where *d*_r_ is the diameter of the inhibitione zone.

CH–CMC	Inhibition Zone	
	*S. aureus*	*E. coli*
0 wt %	-	-
5 wt %	+	+
10 wt %	++	++
15 wt %	++	++
20 wt %	+++	++
